# Zika Virus Alters the Viscosity and Cytokines Profile in Human Colostrum

**DOI:** 10.1155/2019/9020519

**Published:** 2019-11-15

**Authors:** Ocilma B. de Quental, Eduardo L. França, Adenilda C. Honório-França, Tassiane C. Morais, Blanca E. G. Daboin, Italla M. P. Bezerra, Shirley V. Komninakis, Luiz C. de Abreu

**Affiliations:** ^1^Department of Nursing, Faculty of Santa Maria (FSM), Cajazeiras 58900-000, Brazil; ^2^Laboratory of Study Design and Scientific Writing, Department of Morphology and Physiology, Centro Universitário Saúde ABC (FMABC), Santo André, São Paulo 09060-870, Brazil; ^3^Department of Biological and Health Science, Universidade Federal do Mato Grosso (UFMT), Barra do Garças 78600-000, Brazil; ^4^Postgraduate Program in Public Health, School of Public Health, Universidade de São Paulo (USP), São Paulo 01246-904, Brazil; ^5^Postgraduate Program in Public Policies and Local Development, Escola Superior de Ciências da Santa Casa de Misericórdia de Vitória (EMESCAM), Vitória 29027-502, Brazil

## Abstract

The resurgence of cases of Zika virus (ZIKV) infection, accompanied by epidemic of microcephaly in Brazil, has aroused worldwide interest in understanding the biological mechanisms of the virus that allow patient management and the viral dissemination control. Colostrum and human milk are possible sources of virus spread. Therefore, the objective of this study was to analyze the repercussions of ZIKV infection on rheological parameters and inflammatory cytokines of colostrum. The prospective cohort study included 40 puerperal donors of colostrum, divided into 2 groups: control (without ZIKV infection, *n* = 20) and a group infected with ZIKV during the gestational period (*n* = 20). Analyses were performed for the detection of ZIKV by polymerase chain reaction (PCR). In addition to obtaining the rheological parameters and quantification of IL-10 and IL-6 cytokines by flow cytometry, ZIKV and other flaviviruses were not detected in colostrum. However, maternal infection reflected increased viscosity, decreased levels of IL-10, and elevated levels of IL-6. The higher viscosity may represent a mechanical barrier that hinders the spread of the virus. The lower levels of anti-inflammatory mediators and higher inflammatory cytokines may possibly alter the viscosity, and it seems the higher viscosity represents a possible mechanism of adaptation of breastfeeding against a response to ZIKV.

## 1. Introduction

In recent years, Zika virus (ZIKV) infection has become a major public health problem due to the increased incidence of ZIKV contamination and its association with devastating adverse effects such as microcephaly and Guillain-Barré syndrome [1–7].

In 2015, Brazil suffered a large epidemic of microcephaly attributed to congenital infection by ZIKV [5, 8, 9]. It is believed that the virus had a rapid expansion in the country, due to the susceptibility of the population to its vector, the mosquito of the *Aedes* genus [10]. ZIKV infections were not restricted to Brazil; outbreaks and evidence of transmission have appeared in locations throughout the Americas, Africa, and other geographical regions. Around 86 countries and territories reported evidence of ZIKV infection, transmitted by the mosquito [5].

In addition to mosquito bites, it is interesting to note other risk factors that contribute to the increase of ZIKV dissemination potential, such as transmission through sexual relations and maternal-fetal relationship [2, 3, 11] because the virus can be found in several biological fluids in infected individuals, such as in blood, urine, semen, and breast milk [3, 10, 12]. In this context of vertical transmission, questions are raised about the transference of ZIKV to the infant during breastfeeding; however, the data on this topic are still limited [3, 11].

It is known that the host immune response plays an important role in the clinical course of patients with viral infection. Particularly, cytokines may play an essential role in limiting viral spread [13]. Several cytokines that have been found in breast milk and contribute to the development of the child's immune system are related to inflammatory processes [14–16] and metabolic or infectious diseases [17–19], but the effects of maternal infection by ZIKV during gestation on the cytokines present in colostrum have not yet been elucidated.

Immunological and rheological alterations play an important role in some infectious diseases, being attributed an interaction of cytokines with the viscosity for the maintenance of the physicochemical properties of biological fluids [20]. The flow of human milk within the ductal system of the breast is essential to the health and well-being of both mother and child [21]. The viscosity of human milk has been examined in limited studies, but in colostrum from mothers with ZIKV, the rheological properties of human milk have not been studied yet.

It is possible that the ZIKV infections during the gestation could influence the soluble components of human milk impacting its viscosity as well as its proteins, such as cytokines which alters the immunological and rheological parameters of human milk. Thus, the aim of this study was to evaluate the effects of ZIKV infection on rheological parameters and inflammatory cytokines of colostrum during gestation.

## 2. Materials and Methods

### 2.1. Design and Samples

A prospective cohort study was carried out in 2016 and 2017, with 40 women (18-41 years old) who delivered in the public hospital of the State of Paraiba, Northeastern Brazil. Participants donated a colostrum sample, and they were interviewed again at 1 year postpartum by cell phone for data collection about possible child health complications. They were divided into 2 groups according to the presence or absence of infection by ZIKV during their gestational period. The control group (*n* = 20) was composed of women clinically healthy and the ZIKV group (*n* = 20) by puerperae that had ZIKV infection during pregnancy. These women had in their records the confirmation of the diagnosis of ZIKV infection by real-time PCR (polymerase chain reaction) performed by the Central Laboratory of Public Health of the State of Paraiba.

The inclusion criteria of the study were as follows: gestational age at delivery between 37 and 41 6/7 weeks; negative serological reactions for hepatitis, HIV, and syphilis; clinically healthy at delivery; and informed consent form signed. The exclusion criteria were as follows: twin pregnancy; delivery before the 36th week of gestation, and flavivirus or others infections in the postpartum period.

The women were informed about the purpose of the study, and the benefits of this research. The volunteers signed an informed consent form before entering the study, which was approved by the Institutional Committee for Ethics in Research (46643515.0.0000.5421).

### 2.2. Obtaining Colostrum

About 5 mL of colostrum from each woman were collected in sterile plastic tubes, between 48 and 72 hours postpartum. The samples were centrifuged (160x*g*, 4°C) for 10 minutes, which separated the colostrum into three different phases: cell pellet, an intermediate aqueous phase, and an upper fat layer. The aqueous supernatant (colostrum supernatant) was stored at -80°C for subsequent analysis.

The ZIKV group colostrum was analyzed by PCR to detect the presence of viral ribonucleic acid (RNA).

### 2.3. RNA Extraction in Colostrum from ZIKV Group

Viral RNA extraction from 1 mL of colostrum supernatant was performed using Qiamp™ Viral Blood Kit (Qiagen, Hilden, Germany) following the manufacturer's manual. The RNA was eluted in buffer, aliquoted for quantification in fluorimeter, and the remainder stored in -80°C freezer until be used.

### 2.4. Reverse Transcription Reaction

Purified RNA was used for reverse transcription reaction consisting of the transformation of RNA into complementary deoxyribonucleic acid. For this reaction, 5 *μ*L RNA, 150 ng random primers (Invitrogen, Carlsbad, CA, USA), 0.5 mM deoxyribonucleotide triphosphates (dNTPs) (Invitrogen, Carlsbad, CA, USA), and 6 *μ*L deionized water were used. The components were incubated for 5 minutes at 65°C, with subsequent thermal shock on ice. From this, it was added: 1x of First-Strand Buffer (Invitrogen, Carlsbad, CA, USA), 0.005 M DL-dithiothreitol (Invitrogen, Carlsbad, CA, USA), 40 U RNaseOUT™ (Invitrogen, Carlsbad, CA, USA), and 200 U Super Script™ III (Invitrogen, Carlsbad, CA, USA). The reaction was incubated in a thermocycler using the following cycling: 5 minutes at 25°C, 60 minutes at 50°C, and 15 minutes at 70°C.

### 2.5. Real-Time PCR for the Identification of Zika, Chikungunya, and Dengue Viruses “in House”

RNA extracted from the colostrum supernatant was analyzed by real-time PCR (RT-qPCR) using the hydrolysable probes system (TaqMan™) for Zika, chikungunya, and dengue viruses [22, 23]. All RT-qPCR reactions of this research were performed on the 7500 Real-Time PCR system.

### 2.6. Real-Time PCR for the Identification of Zika, Chikungunya, and Dengue Viruses by RT-QPCR Multiplex

RNA extracted from colostrum supernatant was analyzed by Zika, dengue, and chikungunya (ZDC) multiplex RT-PCR assay (Bio-Rad Laboratories, Hercules, CA, USA), following the manufacturer's manual. This kit has all the required controls for a reliable result; in addition, the reverse transcription reaction and real-time PCR are performed in one step.

### 2.7. Real-Time PCR for Flavivirus Identification (Pan-Flavivirus)

It used an RT-qPCR with a system of hydrolysable probes and primers designed to detect different viruses of the *Flavivirus* genus. This detection system was created for the epidemiological surveillance of flaviviruses with speed incessant accuracy [24].

### 2.8. Colostrum Supernatant Rheological Parameters

The rheological parameters were measured using the Modular Compact Rheometer—MCR 102 (Anton Paar™ GmbH, Ostfildern, Germany), according to França et al. [25]. In all experiments, 600 *μ*L of samples were applied to the surface of the plate reader following the removal of excess sample. The readings were performed with a permanent control of gap measurements with TruGap™ in 0.099 mm increments and the measuring cell Toolmaster™ 50. The temperature control was achieved using T-Ready™ and the software Rheoplus V3.61. The graphics were obtained using Rheoplus. For the flow curves and viscosity, established parameters were based on the control of shear stress (*τ*) to 0-5 Pa for upsweep and 5-0 Pa for downward curves. The tests were conducted under isothermal conditions at 37°C, with 60 readings analyzed.

### 2.9. Quantification of Cytokines

The cytokines IL-6 and IL-10 were measured in colostrum supernatant by BD™ Cytometric Bead Array (BD Biosciences, San Jose, CA, USA) according to the manufacturer's manual. A flow cytometer was used for these analyses (BD FACS Calibur™, Biosciences, San Jose, CA, USA). The data were analyzed using the software FCAP Array™ 1.0 (BD Biosciences, San Jose, CA, USA).

### 2.10. Treatment of Colostrum Supernatant with IL-10

To investigate whether the inflammatory process influenced the viscosity of the human colostrum supernatant, the samples were incubated with IL-10, an important anti-inflammation mediator. In all experiments, 580 *μ*L of samples was incubated with 20 *μ*L of cytokine IL-10 (Sigma, St. Louis, USA; final concentration 100 pg/mL) for 1 h at 37°C [20]. This concentration was previously determined by dose-response curve. The colostrum supernatant was used immediately for the rheological analysis.

### 2.11. Statistical Analysis

Statistical analyses were performed with BioEstat® version 5.0 software (Mamirauá Institute, Belém, Brazil). The results were presented as mean (±standard deviation) or amostral number (%). The Shapiro-Wilk normality test was used. The two-way variance analysis (ANOVA) was used by rheological parameters analysis, and one-way variance analysis (ANOVA) was used by cytokines analysis, both followed by Tukey test. Pearson's test was used to describe the correlation between cytokine concentrations and viscosity. Significant differences were considered when *p* < 0.05.

## 3. Results

### 3.1. Subject Characteristics

Women in the control group were not diagnosed with ZIKV infection during pregnancy. While the mothers belonging to the ZIKV group had diagnosis confirmed by PCR test during gestation, but after delivery, no ZIKV was detected in colostrum. Of these, most had infection in the first trimester of gestation and only one case had infection during the third gestational trimester. Babies from both groups had no changes in the head circumference and there were no cases of microcephaly, but posteriorly, six women reported the development of infant health complications (convulsion, neuropsychomotor development delay, and hearing and vision impairment) (Table 1).

### 3.2. Rheological Parameters of the Colostrum Supernatant from Mothers with or without Gestational ZIKV Infection

**Figure 1:**
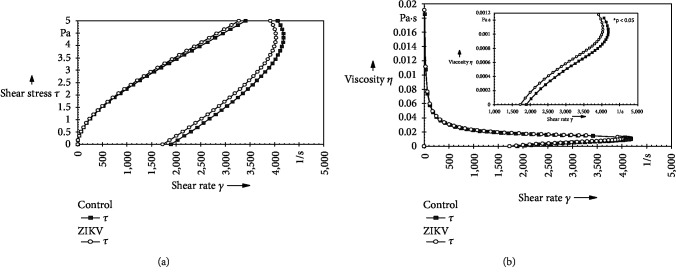
Rheological profile of the colostrum supernatant according with maternal ZIKV infection. (a) Flow curve of the colostrum supernatant from mothers infected or not infected with ZIKV during pregnancy. Viscosity curve (b) of the colostrum supernatant from ZIKV-infected mothers during pregnancy or control group. ^∗^Amplification of the region with a statistical difference (*p* < 0.05); it was assessed by ANOVA (two-way) and Tukey's test.

There was no difference between the groups in the curve of colostrum supernatant flow. The flow curve for both groups started at source, ascended, and was nonlinear (Figure 1(a)).

Colostrum supernatant viscosity analyses at 37°C indicated viscosity was higher in the group of women who had ZIKV infection in the gestational period (Figure 1(b)).

### 3.3. Cytokine Concentrations and Correlations with the Viscosity of Colostrum Supernatant according to Maternal ZIKV Infection.

**Figure 2:**
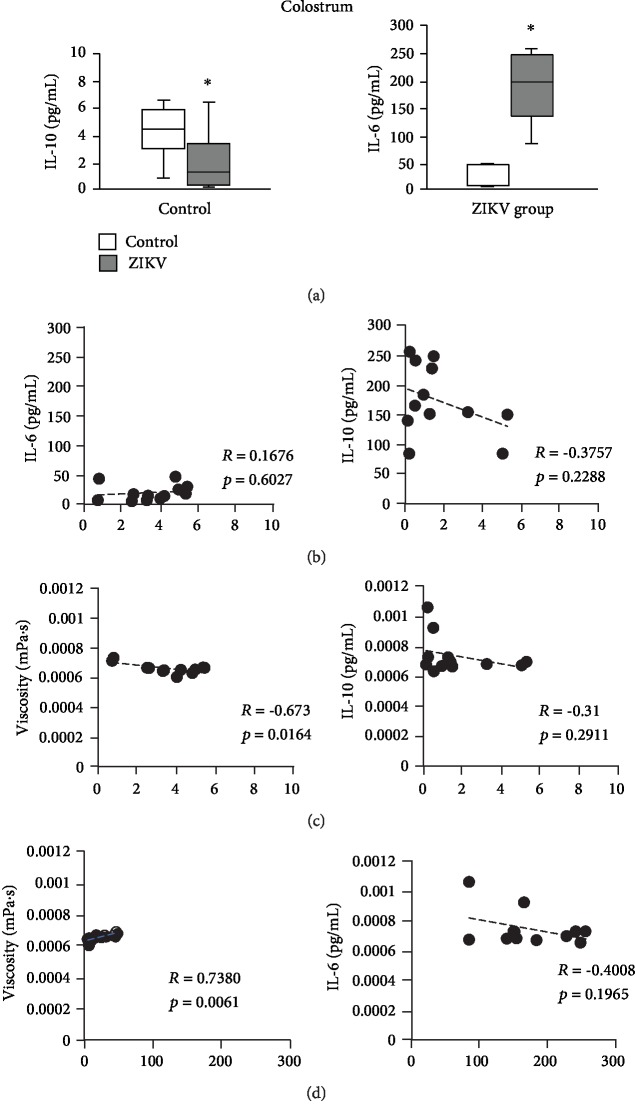
Cytokine levels and correlations with the viscosity of colostrum supernatant according to maternal ZIKV infection. (a) IL-10 and IL-6 levels of the colostrum supernatant from mothers infected or not infected with ZIKV during pregnancy. (b) Pearson's correlation between IL-10 and IL-6 levels for control and ZIKV groups. (c) Pearson's correlation between viscosity and IL-10 levels for both groups. (d) Pearson's correlation between viscosity and IL-6 levels for both groups. ^∗^Statistical difference (*p* < 0.05). ^∗^Statistical difference between the groups (*p* < 0.05); it was assessed by ANOVA and Tukey's test (*n* = 12).

Zika virus infection in the gestational period caused changes in the constituents of IL-10 and IL-6 cytokines in the colostrum supernatant since there was a significant reduction (*p* < 0.05) in IL-10 levels and elevation of IL-6 concentrations in colostrum of women who suffered from ZIKV infection in the gestational period (Figure 2(a)).

Cytokines IL-10 and IL-6 levels correlated with colostrum viscosity only for the control group (*p* < 0.05). IL-10 showed an inversely proportional correlation with the colostrum supernatant viscosity for the control group (*p* < 0.05). While IL-6 showed a correlation directly proportional to the colostrum supernatant viscosity (*p* < 0.05) (Figure 2(d)).

### 3.4. Cytokine Modulation in the Colostrum Supernatant Viscosity from Women with or without ZIKV Infection

**Figure 3:**
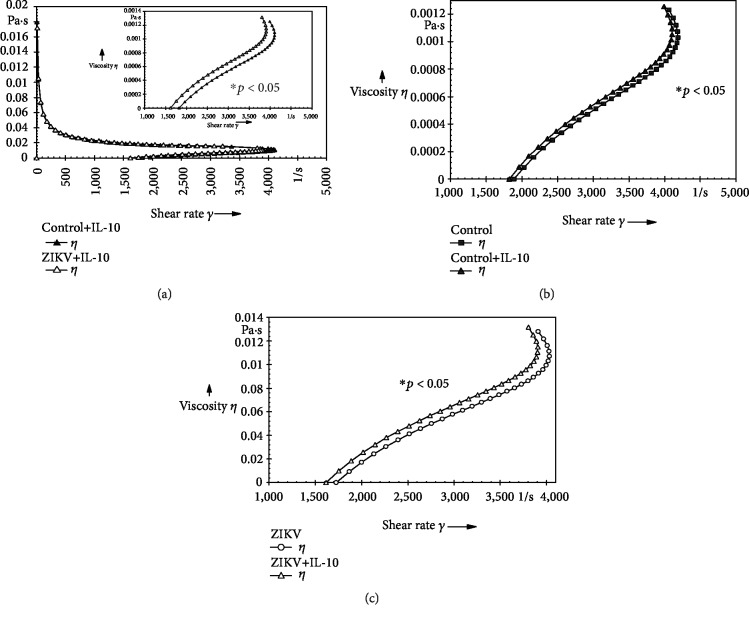
Cytokine modulation in the colostrum supernatant viscosity from individuals with or without ZIKV infection. (a) Viscosity curve of the colostrum supernatant after treatment with IL-10. (b) Viscosity curve of the colostrum supernatant between the control and control plus IL-10 groups. (c) Viscosity curve of the colostrum supernatant between the ZIKV and ZIKV plus IL-10 groups. ^∗^Amplification of the region with a statistical difference (*p* < 0.05); it was assessed by ANOVA (two-way) and Tukey's test.

In order to analyze whether the molecules with action of regulating inflammation cause the alterations in the viscosity, the colostrum supernatant was modulated with the exogenous cytokine IL-10. The results indicated that the exogenous stimulus of IL-10 increases the colostrum supernatant viscosity (*p* < 0.05) for both the control group (Figure 3(b)) and the ZIKV group (Figure 3(c)). However, the regulatory cytokine IL-10 was not enough to compensate the changes reflected in colostrum viscosity for the ZIKV group (Figure 3(a)) (*p* > 0.05).

## 4. Discussion

Human colostrum is a unique biofluid; its flow is essential for both mother and infant. It is composed of soluble and immunoprotective elements that protect the newborn from a variety of pathogenic microorganisms [19, 25–28].

In cases of ZIKV infection during the gestation, the mother develops milder symptoms, but in the fetus, the virus may cause growth restriction, a spectrum of central nervous system abnormalities, or even fetal death [10]. As a result, congenital infection may not be clinically detected during gestation, but adverse effects may appear in the postgestation period and during breastfeeding [29]. Accordingly, our results revealed change in cytokines and viscosity in human colostrum of mothers who had ZIKV infection during their gestational period.

In Brazil, the diagnosis of ZIKV infection depends on the identification of the virus by RT-PCR performed during the acute period of infection [10]. The virus is detectable in the blood during the acute viraemia period, and it is eliminated through the urine, usually for more than 10 days [30]. While in biological fluids such as semen, ZIKV is present up to 117 days after the onset of symptoms [12]. It also has been found in colostrum and human milk [11, 31, 32].

In this study, we did not observe the presence of ZIKV RNA in colostrum of mothers who had the viral infection during the gestational period. Possibly, the nondetection of ZIKV in these colostrum samples was due to the fact that women donors were out of the viraemia period of the disease. In the studied population, ZIKV infection occurred mostly within the first three months of gestation.

The gestational week of maternal infection may influence the presence or absence of ZIKV in colostrum and breast milk. In this sense, a study describes the case of pregnant women with Zika infection at the 36th week of gestation, that even 30 days after the onset of signs and symptoms, ZIVK was present in colostrum (2.44 × 10^6^ copies/mL) and in the breast milk (9 days postpartum and 216,000 copies/mL), with no signs of infection in the infant [32].

It is worth remembering that the neonate is very susceptible to infections by bacteria and viruses [33]. Therefore, it is essential that the child be breastfed. Breastfeeding promotes the passage of maternal antibodies to the infant and is an additional protection in cases of infection until the baby's immune system is developed [34–38]. In addition human milk to directly modulating the baby's immunological development, due to the cytokine profile in its constituents [14, 39].

Possibly, cytokines play an important role in the replication and dissemination of ZIKV. Therefore, their concentrations in biological fluids from viral infections may be altered [12].

Changes in the cytokine profile, due to inflammatory processes in the presence of microorganisms, may alter the viscosity of biological fluids. Therefore, viscosity analysis is a tool to assist the clinical diagnosis of diseases [20] and, in addition, to represent a fundamental physical property for the quality indicator of human milk [40].

It is of great relevance that the fact that among biological fluids, breastfeeding represents a less efficient transmission pathway than other body fluids [11]. The outcomes of studies that detected infectious ZIKV particles in colostrum and human milk, without conclusive cases of infant infection are instigating [11, 32]. Therefore, we assume that maternal infection with ZIKV, during the gestational period, causes changes in human colostrum, by mechanisms involved in altering the viscosity and in the profile of inflammatory cytokines. Thus, benefiting the health of the infant against possible negative impacts caused by ZIKV.

To explore this hypothesis, the viscosity was analyzed by rheology. The rheological parameters of the colostrum supernatant curve, independent of maternal infection by ZIKV, did not show ideal liquid characteristics. The lack of overlapping of the upward and downward curves leads to the formation of the hysteresis area that defines the magnitude of the thixotropic property in the sample [25].

In this research, the thixotropy of human colostrum was affected by maternal infection from ZIKV in the gestational period (independent of gestational age), resulting in a significant difference in viscosity between the analyzed groups. This fact suggests that the increase in viscosity, described in the colostrum of ZIKV group, represents a protection, which can serve as a mechanical barrier, developed by the mammary gland, that it makes difficult the dissemination of ZIKV.

Given its originality, it is difficult to compare rheological parameters of human colostrum or biological fluids from individuals who had ZIKV with others study. It is known that the use of bioactives may alter the viscosity of the biological fluid of the affected group and provide strategic pathways to manage the disease [20, 25]. These bioactives usually modulate the inflammatory process caused by the infection [20]. The development and resolution of the inflammatory process are regulated by a complex network of cytokines that have pro- and anti-inflammatory effects. The effective action of a proinflammatory cytokine depends on the synergy with other inflammatory cytokines and antagonists (anti-inflammatory) that are usually elevated at the infection area [41]. In this process, the cytokines IL-10 and IL-6 are fundamental and they are present in colostrum and breast milk [14, 39].

The cytokine IL-10 is mainly known for its regulatory activity and for inhibiting the proinflammatory responses of innate and adaptive immunity, thus preventing tissue damage due to exacerbated immune response [42], while IL-6 is responsible for activating responses of cellular components of the innate immune system and coordinates lymphocyte proliferation [43]. It is generally elevated in most inflammatory stages but eventually exhibits anti-inflammatory activity. It is also involved in the regulation of metabolic, regenerative, and neural processes [44].

In this study, a significant reduction in IL-10 levels and a significant increase in IL-6 concentrations were observed as a function of maternal infection by ZIKV in the gestational period. It emphasizes that both groups were clinically healthy at the time of colostrum collection; thus, this change in colostrum inflammatory cytokines does not refer to other diseases. In the scientific literature, there are gaps in the profile of colostrum and human milk cytokines in patients with ZIKV infection.

Study with blood samples described an increase in IL-6 levels, without changes in IL-10 levels, for the group of individuals infected by ZIKV, when compared to noninfected individuals [13]. While in the semen, patients infected with ZIKV had higher concentrations of IL-6 and IL-10 than the control, in order to evidence the persistent inflammation since the beginning of the infection [12]. Levels of IL-10 or IL-6 may be variable and may increase or decrease in relation to the control, according to the type of biological fluid, virus strain, and days after the onset of symptoms [12, 43].

In this study, no significant correlation was found between IL-10 levels and IL-6 levels in colostrum. However, these cytokines showed a correlation with viscosity for the control group.

It was observed that, in healthy subjects, IL-10 and IL-6 cytokine concentrations were, respectively, inversely or directly correlated with viscosity. It is indicative that in healthy individuals, a balance occurs between inflammation mediators (IL-10 and IL-6) to maintain colostrum viscosity. The same did not happen for the ZIKV group, suggesting that the elevation of colostrum viscosity for this group was not directly due to the elevation of IL-6. Possibly, the change in the cytokine profile triggered the release of other biological components that reflected the increase in viscosity.

It is likely that the changes observed in colostrum IL-10 and IL-6 cytokines reflect protective mechanisms of breastfeeding since IL-10, in the viral response, can impair the initiation of T cells in the early stages of adaptive immunity, which can be used as a mechanism of scape that promotes viral persistence [42].

Another interesting fact is that IL-6 induces significant increases in secretory Immunoglobulin A (SIgA) [45, 46], which is present in human colostrum [36] and is the most abundant immunoglobulin found in the lumen of the human intestine. It represents, therefore, the first line of defense of intestinal protection against pathogens [36, 47].

It is also essential to state that the action of IL-10 under the viscosity of colostrum was dependent on its concentration, whereas, in endogenous concentrations in healthy individuals, it has an inverse correlation with viscosity and while used at a higher level (exogenous stimulation), it increased viscosity in both groups. Thus, we can infer that the use of components with anti-inflammatory action in puerperae should be used with caution, because they interfere in the alteration of cytokine levels that provide colostrum with a feasible biological protection against ZIKV infection to the infant.

Another important point to debate is that between the mother and infant dyad, there is an interaction mechanism that can alter immunological components of breast milk due to infections in the infant. The human milk promotes the process inflammatory regulation with the aim of conferring additional immunological support to the infant [48]. In addition, the human milk and the infant's saliva appear to represent a biochemical synergism which boosts early baby's innate immunity [49]. So, the detection alterations in the IL-10 and IL-6 cytokines levels on colostrum of ZIKV group may be related with a baby's health status. Newborns at the time of delivery did not have health complications. It is possible that inflammatory process resulting from gestational ZIKV infection trigger consequences for the fetus that not were detected by standard routine initial examinations but that may appear at a later period. This interaction could not be evaluated due to the limitation of this study that does not cover cord blood analysis.

New research approaches should be conducted to investigate rheological properties and action of the mammary gland as an organ of the immune system in mothers with ZIKV infection aiming to understand the mechanisms of milk flow and of nutritional and immunological components.

The results of the present study reinforce the importance of a rigid control of ZIKV infection during pregnancy in order to maintain the normal flow of milk and that the immunity components are properly provided. Despite the abnormalities in rheological and immunological parameters, women that were infected with ZIKV should be strongly encouraged to breastfeed their children, since the presence of the virus in the secretion was not evidenced. In addition to being an excellent source of food for newborns, breast milk decreases the high rates of maternal and infant complications.

## 5. Conclusions

Maternal infection by ZIKV during gestation triggers increased viscosity and changes in IL-10 and IL-6 cytokine levels when compared to colostrum in noninfected women. The lower levels of anti-inflammatory mediators and higher inflammatory cytokines may possibly alter the viscosity and may represent a possible mechanism of adaptation of breastfeeding against a response to ZIKV.

## Figures and Tables

**Table 1:** Maternal and neonate parameters according to maternal gestational ZIKV infection.

Maternal and child parameters	Control (*n* = 20)	ZIKV (*n* = 20)
*Age (years)*	26.10 (±4.91)	25.95 (±7.24)
*Signs and symptoms of gestational Zika*	00 (0.00%)	20 (100.00%)
*RT-PCR (serum) (%)—ZIKV+*	—	20 (100.00%)
*Gestational age at ZIKV infection (trimester)*		
1 trimester	—	13 (65.00%)
2 trimester	—	6 (30.00%)
3 trimester	—	1 (5.00%)
*Gestational age at delivery (weeks)*	38.95 (±1.00)	39.65 (±0.99)
*Infant sex*		
Female (%)	11 (55.00%)	11 (55.00%)
Male (%)	9 (45.00%)	9 (45.00%)
*Birth weight (g)*	3375.50(±444.89)	3356.50 (±407.43)
*Birth height (cm)*	45.50 (±3.46)	48.00 (±3.52)
*Brain perimeter (cm)*	34.20(±0.89)	33.76 (±0.66)
*Microcephaly*	00 (0.00%)	00 (0.00%)
*Infant health complications (outcomes at 1* year *postpartum)*	—	
Hearing impairment	—	2 (10.00%)
Vision impairment	—	1 (5.00%)
Convulsion	—	1 (5.00%)
Neuropsychomotor development delay	—	2 (10.00%)
*ZIKA IgG/IgM (colostrum)—negative (%)*	20 (100.00%)	—
*PCR (colostrum)—ZIKV and flavivirus—(%)*	—	20 (100.00%)

Maternal and neonatal data are shown as mean (±SD) or amostral number (%).

## Data Availability

The data used to support the findings of this study are available from the corresponding author upon request.
